# Cuticular pheromones stimulate hygienic behavior in the honey bee (*Apis mellifera*)

**DOI:** 10.1038/s41598-020-64144-8

**Published:** 2020-04-28

**Authors:** Kaira M. Wagoner, Jocelyn G. Millar, Coby Schal, Olav Rueppell

**Affiliations:** 1 Biology Department, University of North Carolina at Greensboro, Greensboro, USA; 2 Department of Entomology, University of California, Riverside, USA; 3 Department of Entomology & Plant Pathology, North Carolina State University, Raleigh, USA

**Keywords:** Biochemistry, Biological techniques, Chemical biology, Ecology

## Abstract

The health of western honey bee (*Apis mellifera*) colonies is challenged by the parasitic mite *Varroa destructor* and the numerous harmful pathogens it vectors. Selective breeding for the naturally occurring social immune trait “hygienic behavior” has emerged as one sustainable approach to reducing the mites’ impact on honey bees. To expand our understanding of hygienic triggers and improve hygienic selection tools, we tested the hypothesis that the cuticular compounds (*Z*)-10-tritriacontene and (*Z*)-6-pentadecene, previously associated with unhealthy honey bee brood and/or brood targeted for hygiene, are triggers of honey bee hygienic behavior independent of brood health. In support of our hypothesis, application of synthetic (*Z*)-10-tritriacontene and (*Z*)-6-pentadecene onto brood and brood cell caps significantly increased hygienic behavior compared to application of similarly structured hydrocarbon controls (*Z*)-16-dotriacontene and (*Z*)-7-pentadecene. Furthermore, we demonstrate a significant positive correlation between colony-level hygienic responses to (*Z*)-10-tritriacontene and the traditional freeze-killed brood assay for selection of hygienic honey bee stocks. These results confirm biological activity of (*Z*)-6-pentadecene and reveal (*Z*)-10-tritriacontene as a novel hygiene trigger. They also support development of improved tools for honey bee colony monitoring and hygienic selection, and thus may accelerate development of honey bee stocks with greater resistance to *Varroa* and associated pathogens.

## Introduction

As with other social insects, honey bees are highly susceptible to the horizontal spread of infectious diseases due to close contact, high coefficients of relatedness, and high frequency of social interactions among individuals within a colony^1–3^. Movement of parasites between bees within a colony can facilitate the spread of honey bee diseases^2^ because ectoparasites such as the mite *Varroa destructor*, hereafter *Varroa*, vector numerous honey bee pathogens^4,5^. In addition, *Varroa* can amplify viral loads^6–8^, alter viral strain diversity^8^, and affect virus virulence^9,10^. In the western honey bee (*Apis mellifera*), the spread and proliferation of harmful pathogens such as Deformed Wing Virus (DWV) is especially problematic considering honey bees have fewer immune-related genes than non-social insects^11^.

Combined with susceptibility to and spread of honey bee parasites and pathogens^12–14^, numerous anthropogenic threats such as pesticide exposure and poor nutrition have led to a shortage of colonies relative to an increasing global demand for crop pollinators^15–18^. Following pollinator population trends in much of the Northern Hemisphere^19–21^, managed honey bee colonies in the United States have declined more than 50% over the last six decades, from around 5.9 million colonies in 1947 to around 2.7 million colonies in 2015^22,23^. Early declines were likely due to reduced public demand for honey after the end of World War II, while the spread of pests and pathogens are responsible for more recent spikes in annual colony losses^22^. Each year between 2012 and 2016, the average total annual colony losses in the United States exceeded 34%, and overwintering loss rates were consistently higher than levels deemed acceptable by beekeepers^23–26^. Among multiple, interacting risks to honey bee health, *Varroa* are considered the most severe threat to modern apiculture because their recent and rapid global expansion has resulted in increased beekeeping costs and decreased honey bee health, leading to decreases in the number of active beekeepers^27^ and colony overwintering survival^21,28^.

Despite substantial evidence of a need to control *Varroa*, no satisfactory solution has been discovered^29^. Miticides used to reduce *Varroa* populations are harmful to bees and are only temporarily effective due to mites’ rapid evolution of resistance^27,30^. Alternative control strategies, such as physical mite removal and use of essential oils, have many limitations that compromise efficacy, including increased labor for beekeepers (and thus lack of adoption), sensitivity to fluctuations in ambient temperature, and minimal differences between lethal doses for mites and honey bees^29,31^. Thus, one of the most promising strategies to combat *Varroa* is the selective breeding of disease-resistant honey bees, including Minnesota Hygienic (HYG) and *Varroa* Sensitive Hygienic (VSH) stocks.

Hygienic behavior is the detection, uncapping, and removal of diseased or parasite-infested brood from the colony^32,33^, and is one of the resistance mechanisms of *Varroa*’s original host, the eastern honey bee *Apis cerana*^34^. It also occurs naturally in *A. mellifera* at a low frequency and is most commonly observed in worker bees aged 15 to 20 days^35^. HYG bees are selected based on their hygienic removal of freeze-killed brood^36^, whereas VSH bees are selected based on their apparent suppression of mite reproduction^37^. Although both of these stocks have reduced pathogen loads compared to unselected (UNS) colonies^32,37,38^, the process of selecting for suppression of mite reproduction is time-consuming and other interventions, including miticides, are still needed to control severe mite infestations in HYG colonies^39,40^. Thus, there is need for an optimized assay that is rapid, user-friendly, and specific to honey bee pests and pathogens.

Insect cuticles are coated with various lipids, including hydrocarbons, wax esters, glycerides, free fatty acids, sterols, aldehydes, and alcohols^41,42^. These compounds reduce water loss and facilitate inter- and intraspecific communication^43^. In honey bees, cuticular compounds are typically dominated by hydrocarbons, specifically alkanes, alkenes, and methylalkanes^44–46^. Previous studies have provided evidence of significant effects of DWV and *Varroa* infestation on the composition of cuticular hydrocarbons in honey bee brood^47–50^ and have linked such quantitative changes to hygienic removal^49,50^. Specifically, parasitized and DWV-infected brood have been associated with higher proportions of several unsaturated hydrocarbons including pentadecene, hentriacontene, and tritriacontene^47,49–52^, and specific isomers of these alkenes, including (*Z*)-6-pentadecene (Z6-C_15_), (*Z*)-8-hentriacontene (Z8-C_31_), and (*Z*)-10-tritriacontene (Z10-C_33_; subsequent abbreviations conform to this pattern), have been associated with brood targeted with hygienic behavior^49,50^. However, with one notable exception^49^, current evidence for relationships between brood hydrocarbons and honey bee hygiene is either correlative or chemically undefined (i.e., cell treatment with brood extracts), suggesting the need for analysis of the effects of direct application of individual chemicals of interest to honey bee brood cells.

In this study we investigated hygienic effects of two hydrocarbons previously associated with stressed honey bee brood and hygienic behavior. Based on work by Nazzi *et al*.^49^ and our own work^50^, we tested the hypothesis that Z6-C_15_ and Z10-C_33_ are triggers of honey bee hygienic behavior, independent of brood health. These two hydrocarbons were selected based on their associations with honey bee stressors (parasites and viruses) and hygiene in previous studies as described above^49,50^. Additionally, these chemicals were chosen based on their distinct volatilities in typical colony conditions, as high and low volatility may be important for attracting hygienic workers to a general area, and pinpointing problematic cells, respectively^53^. We predicted that honey bee hygienic responses to Z10-C_33_ and Z6-C_15_ application to honey bee brood and brood cell caps would 1) be greater than hygienic responses toward hydrocarbon controls of similar structure, 2) exhibit a dose-response relationship, and 3) be positively correlated with honey bee responses in the freeze-killed brood (FKB) assay.

## Results

### Hygienic response to treatment of pupae with Z10-C_33_

**Figure 1:**
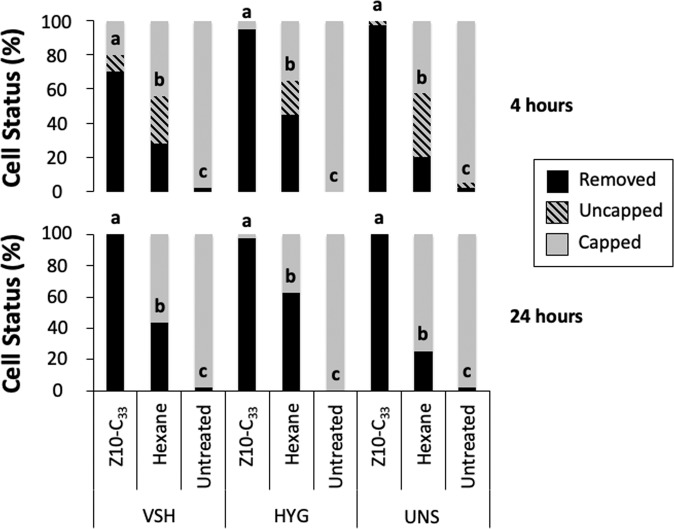
Hygienic responses to pupae treated with 1 μL of 1% Z10-C_33_ in hexane, or 1 μL hexane, or left as untreated controls, in three colonies from different breeding backgrounds. Letters indicate significant differences in cell status between treatments for each colony. Compared to either control (hexane or untreated), significantly more cells treated with Z10-C_33_ were uncapped and the pupae removed, at both 4 and 24 h post treatment, in all three colonies tested. Hexane treatment also elicited significantly greater hygienic responses than untreated controls at both time points and in all three colonies.

Treatment of pupae with 1 μL of 1% Z10-C_33_ in hexane had a significant effect on hygienic response in VSH (X^2^ = 64.25, d.f. = 2, p < 0.001), HYG (X^2^ = 75.80, d.f. = 2, p < 0.001), and UNS (X^2^ = 72.97, d.f. = 2, p < 0.001) colonies at 4 h, and in VSH (X^2^ = 96.76, d.f. = 2, p < 0.001), HYG (X^2^ = 78.42, d.f. = 2, p < 0.001), and UNS (X^2^ = 85.32, d.f. = 2, p < 0.001), colonies at 24 h (Fig. 1). Bonferroni-corrected post-hoc analyses indicated that Z10-C_33_, hexane, and control treatments were significantly different from one another in each colony and at each time point. Lower numbers of manipulated (removed or uncapped) cells at 24 h than 4 h provides evidence of recapping (Fig. 1).

### Development after treatment of pupae with Z10-C_33_

**Figure 2:**
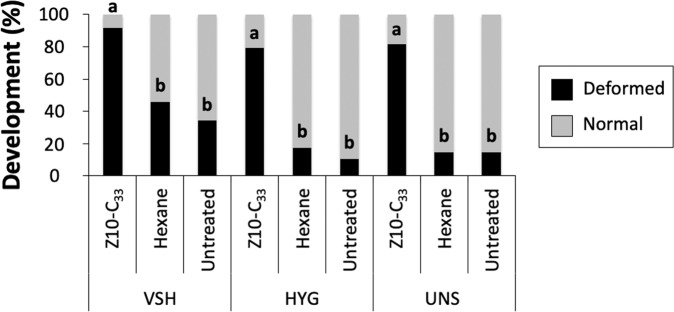
Developmental status of *in vitro* reared pupae from three colonies with different breeding backgrounds treated with 1 μL of 1% Z10-C_33_ in hexane, or 1 μL hexane, or left untreated as controls. Letters indicate significant differences in developmental status between treatments for each colony. Treatment had a significant effect on the number of deformed bees at the time of expected adult emergence in VSH, HYG, and UNS colonies. Compared to either control, significantly more pupae treated with Z10-C_33_ exhibited deformities at the time of expected adult emergence in all three colonies tested.

Direct treatment of pupae with 1 μL of 1% Z10-C_33_ in hexane had a significant effect on development in brood from VSH (X^2^ = 26.13, d.f. = 2, p < 0.001), HYG (X^2^ = 36.48, d.f. = 2, p < 0.001), and UNS (X^2^ = 34.31, d.f. = 2, p < 0.001) colonies (Fig. 2). Post-hoc analyses indicated that for VSH, HYG, and UNS colonies, abnormal development was significantly higher for Z10-C33-treated pupae than for either the hexane or untreated control treatments (Fig. 2).

### Hygienic response to treatment of wax caps with hydrocarbons

**Figure 3:**
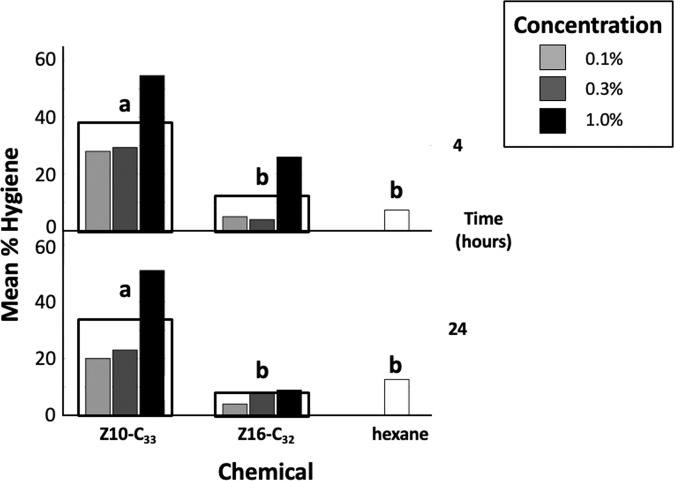
Hygienic responses at 4 and 24 h post treatment of brood cell caps with 0.1%, 0.3%, or 1% Z10-C_33_ in hexane, 0.1%, 0.3%, or 1% Z16-C_32_ in hexane_,_ or hexane. Hollow rectangles represent mean hygienic response over the three concentrations tested. Letters indicate significant differences in mean hygienic responses between treatment groups for all concentrations tested. Compared to either control, a significantly greater hygienic response was elicited by Z10-C_33_ at both 4 and 24 h after treatment.

Z10-C_33_ application to cell caps compared to control applications (Fig. 3), had a significant effect on hygienic behavior at both 4 h (F_1,10_ = 9.34, p = 0.01) and 24 h (F_1,10_ = 30.42, p < 0.001). Post-hoc tests indicated that hygienic response to Z10-C_33_ was significantly higher than to Z16-C_32_ and hexane at 4 h (p = 0.03 and p = 0.04, respectively), and 24 h (p < 0.001 and p = 0.007, respectively). There were no significant differences between effects of Z16-C_32_ and hexane at 4 h (p > 0.99) or 24 h (p > 0.99). The effect of chemical concentration on hygienic removal was marginally significant at 4 h (F_2,10_ = 3.68, p = 0.06) and statistically significant at 24 h (F_2,10_ = 10.90, p = 0.003). At 24 h, the highest hydrocarbon concentration (1%) elicited significantly greater hygienic response than lower concentrations (p = 0.01, p = 0.003, and 0.007 for 0.3%, 0.1%, and 0% (hexane), respectively), but the effects on hygiene were not different between the two lower concentrations (p > 0.99 for each comparison). Interaction effects between chemical type and concentration were not significant at either 4 or 24 h (F_2,10_ = 0.038, p = 0.96 and F_2,10_ = 2.87, p = 0.10, respectively). Lower numbers of manipulated cells at 24 h than 4 h provides evidence of recapping.

**Figure 4:**
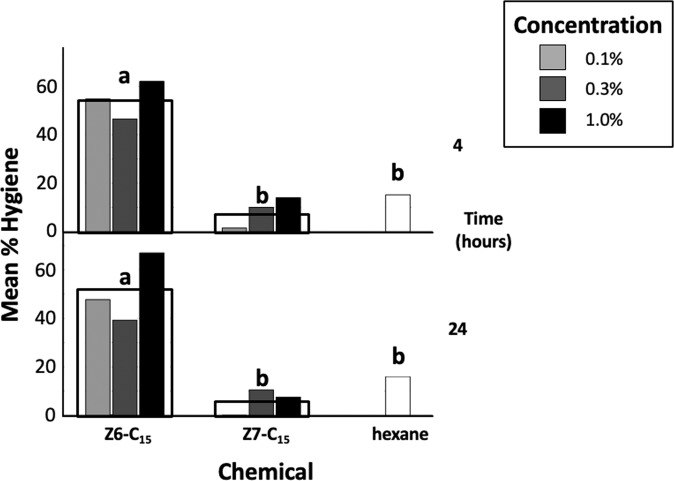
Hygienic responses at 4 and 24 h after treatment of brood cell caps with 0.1%, 0.3%, or 1% Z6-C_15_ in hexane, 0.1%, 0.3%, or 1% Z7-C_15_ in hexane_,_ or hexane. Hollow rectangles represent mean hygienic response over the three concentrations tested. Letters indicate significant differences in mean hygienic responses between treatment groups for all concentrations tested. Compared to either control, Z6-C_15_ elicited significantly greater hygienic responses at both 4 and 24 h after treatment.

Z6-C_15_ applied to cell caps significantly affected hygienic behavior at both 4 h (F_1,16_ = 44.89, p < 0.001) and 24 h (F_1,16_ = 20.04, p < 0.001) compared to the Z7-C_15_ control (Fig. 4). In post-hoc tests, the effect of Z6-C_15_ on removal was significantly different from that of Z7-C_15_ and hexane at 4 h (p < 0.001 and p = 0.004, respectively), and 24 h (p = 0.001 and p = 0.01, respectively). There were no significant differences between effects of Z7-C_15_ and hexane at 4 h (p > 0.99) or 24 h (p > 0.99). The effect of chemical concentration on hygiene was not significant at 4 h (F_2,16_ = 0.49, p = 0.62) or 24 h (F_2,16_ = 0.72, p = 0.50). Interaction effects between chemical type and concentration were not significant at either 4 or 24 h (F_2,16_ = 0.28, p = 0.76 and F_2,16_ = 0.78, p = 0.47, respectively). Lower numbers of manipulated cells at 24 h than 4 h provides evidence of recapping.

### Effects of wax cap treatment on development

**Figure 5:**
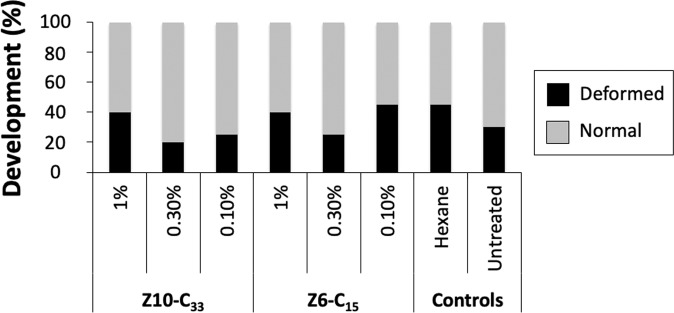
Developmental status of *in vitro* reared pupae from cells whose caps were treated with hexane solutions of Z10-C_33_ or Z6-C_15_, or hexane, or that remained untreated as controls. Treatment had no significant effect on the number of deformed bees at the time of expected adult emergence.

Treatment of wax caps with Z10-C_33_ and Z6-C_15_ had no significant effect on pupal development (X^2^ = 6.06, d.f. = 7, p = 0.53) (Fig. 5).

### Comparison of freeze-killed brood (FKB) assay and Z10-C_33_-treatment assay

**Figure 6:**
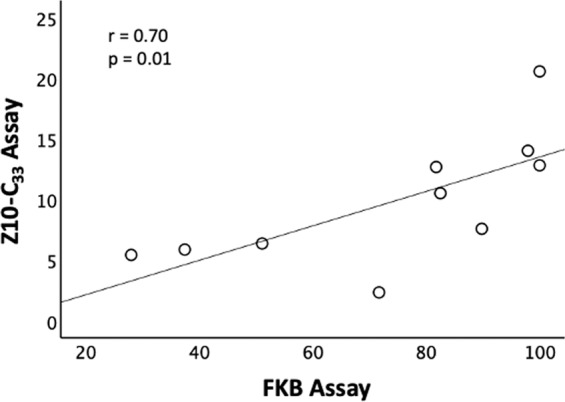
Correlation between scores for the FKB Assay (freeze-killed brood assay, scored for complete removal after 24 h) and Z10-C_33_ Assay (scored as uncapping or removal after 24 h) for 10 colonies of different origins. A significant positive correlation between colony responses to the two assays was observed.

A significant positive correlation (r = 0.70, n = 10, p = 0.01) was found between Z10-C_33_ and FKB assays tested in 10 colonies, with a greater range in responses in the FKB assay (Fig. 6).

## Discussion

Two major types of chemical cues and signals can guide honey bee workers to perform hygienic behavior. First, compounds not naturally found on honey bee brood may be perceived as “foreign” cues by workers, and could elicit some form of uncapping and/or removal behavior when detected in brood cells or on brood cell caps. This form of chemosensory processing is similar to the recognition of foreign invaders in social insect colonies^41,54–56^. Hygienic behavior may also be stimulated by intraspecific signals (pheromones) through changes in amounts and ratios of naturally occurring native chemicals. This form of chemosensory processing is similar to the recognition of nestmates vs. conspecific non-nestmates in social insect colonies^54,57–59^. Notably, cuticular hydrocarbons play important functions in both intra- and interspecific recognition in social insects^60–62^. Our and previous results indicate that the hydrocarbons Z10-C_33_ and Z6-C_15_, when applied to brood or capped brood cells, induce hygienic behavior. In contrast, the structurally similar hydrocarbons Z16-C_32_ and Z7-C_15_, which have not been associated with honey bee brood, elicited significantly less hygienic behavior. Thus, the chemical trigger for hygienic behavior is more similar to intraspecific communication than to a general recognition of a foreign stimulus. Furthermore, we show that hygienic responses to Z10-C_33_ and Z6-C_15_ may be useful as indicators of hygienic behavior at the colony level. Though previous studies have involved application of synthetic brood chemicals directly to pupae^49^ and extracts of brood signals to brood cell caps^50^, to our knowledge, this is the first study to apply synthetic versions of honey bee brood compounds to brood cell caps to induce hygienic behavior. Therefore, results from this study not only confirm the biological activity of two cuticular hydrocarbons in triggering hygienic behavior towards otherwise healthy brood, but may also be useful in the development of improved tools for hygienic selection of pest- and disease-resistant honey bees, although further research related to quantitative and interactive effects of chemicals on hygienic behavior are needed.

Both Z10-C_33_ and C_15_ (isomers unidentified) occur naturally on honey bee cuticles, are found in higher quantities on *Varroa*-infested brood, and have been linked to hygienic behavior^47,49,50^. The experimental results support our hypothesis that Z10-C_33_ and Z6-C_15_ are triggers of honey bee hygienic behavior independent of brood health. Direct treatment of pupae with Z10-C_33_, and to a lesser extent hexane, elicited hygienic behavior in VSH, HYG, and UNS colonies. While these results are similar to those previously reported for Z6-C_15_-treated brood^49^, *in vitro* rearing indicated that increased hygienic behavior might have been partially due to a detrimental effect of direct exposure of brood to Z10-C_33_.

Hygienic behavior has been likened to programmed cell death^63^ or “social apoptosis”^64^, with the idea that removal of unhealthy individuals may improve overall colony health. Although the effect of Z10-C_33_ on brood susceptibility at quantities naturally produced by brood was not tested here, the considerable detrimental effect of Z10-C_33_ on pupal development combined with the lack of a hexane effect suggests that brood susceptibility to natural hygienic signals could play a role in hygiene at the colony level. It is unclear whether the hygienic signal could represent a coopted apoptosis mechanism^64^ but this idea is consistent with recent findings that brood signaling differs by honey bee stock^50^, and that high brood susceptibility at the individual level may confer resistance to *Varroa* at the colony level^64^. Indeed, further studies regarding relationships among honey bee stock, quantitative brood signaling, and brood susceptibility are needed to clarify whether developmental interference caused by naturally produced brood signals plays a role in the apoptotic induction of hygienic behavior.

Because we were primarily interested in the function of applicable chemical signals (independent of induced mortality and/or developmental abnormalities), we shifted to treating brood cell caps rather than pupae directly. We found clear evidence that the hydrocarbons Z10-C_33_ and Z6-C_15_, previously associated with *Varroa* and DWV-stressed brood, when applied to brood cell caps, elicited hygienic behavior. This effect was specific to these particular compounds because the structurally similar chemicals Z16-C_32_ and Z7-C_15_, which have not previously been associated with honey bee brood, did not have a comparable effect. Because development of brood under Z10-C_33_ and Z6-C_15_ treated caps was not different from that of brood under hexane treated and untreated caps, we conclude that the hygienic behavior observed was not a result of brood abnormality related to cap treatment. Differential responses to Z10-C_33_ and Z6-C_15_ compared with structurally similar hydrocarbon controls may be related to two chemosensory mechanisms. First, control hydrocarbons may be detected but ignored as irrelevant. Some, albeit low, responses to high concentrations of control hydrocarbons support this idea. Second, honey bees may possess receptors specifically tuned to Z10-C_33_ and Z6-C_15_, whereas they may be functionally anosmic to the structurally related but “non-natural” Z16-C_32_ and Z7-C_15_. Nazzi *et al*.^49^ tested honey bee responses to brood treated with Z6-, Z7-, and Z8-C_15_ isomers, and found Z6-C_15_ to be the most effective at triggering honey bee hygienic behavior. Additional structure-activity studies that expand analyses of bioactive natural hydrocarbons to multiple colonies could improve the conclusiveness of these findings, and provide critical data to discriminate between these two hypotheses.

Finally, we identified a significant positive correlation between hygienic responses to Z10-C_33_ and to FKB across 10 honey bee colonies. This suggests that hygienic response to Z10-C_33_ may be related to hygienic response to the similarly non-volatile necromone oleic acid, previously associated with FKB^53^. While our findings suggest that chemical hygiene triggers may be useful in measuring colony hygiene level, colony response 24 h after treatment with Z10-C_33_ was relatively low and had a smaller range than response to FKB. Accordingly, and because natural signals likely involve mixtures rather than isolated compounds^50^, further studies involving shorter assay times that prevent misclassification of recapped cells, higher chemical concentrations, additional compounds, and mixtures of chemical stimuli should be conducted to optimize development of an improved assay for measuring hygienic behavior specific to *Varroa* and brood diseases.

Together, our findings provide support for the hypothesis that Z10-C_33_ and Z6-C_15_ are triggers of honey bee hygienic behavior independent of brood health. From a practical viewpoint, our results showed that synthetically produced compounds can be applied to capped brood cells to elicit honey bee hygienic behavior. This approach could be developed as a tool for evaluation of honey bee hygiene at the colony level. Such an assay may be useful for the improvement of selective breeding, because colony responses to actual brood stress signals may rely on different mechanisms or olfactory sensitivities than selection based on brood killed by freezing in the FKB assay^65–67^. Thus, semiochemical assays may be better suited to distinguish colonies with enhanced disease- and pest-resistance, facilitating honey bee management decisions such as which colonies may benefit most from chemical treatment to manage *Varroa*, or which breeders provide the most hygienic queens. A semiochemical hygiene assay may also be more rapid, and more beekeeper-friendly than current selection methods which require killing, infestation, and/or meticulous inspection of brood. Consequently, testing and breeding for hygienic behavior may become more widespread. Improved honey bee breeding and management decisions have potential to sustainably improve honey bee health, reducing the risks associated with many current *Varroa* management practices, such as evolution of pest and pathogen resistance, and contamination of commercial honey and beeswax. Thus, although further development is needed, our findings suggest that the exploitation of intrinsic signals associated with honey bee health may provide new tools and strategies of benefit to queen breeders, commercial beekeepers, farmers, and consumers.

## Methods

A recent study linked Z10-C_33_ to *Varroa*-infested, DWV-infected, and hygiene-targeted honey bee brood^50^. Given the structural similarity of Z10-C_33_ to Z6-C_15_, previously linked to hygienic removal^49^, we decided to investigate the effectiveness of Z10-C_33_ and Z6-C_15_ in triggering hygienic behavior. For this purpose, *Varroa*-Sensitive Hygienic (VSH), Minnesota Hygienic (HYG), and unselected control (UNS) honey bee colonies were established at the University of North Carolina at Greensboro apiary in the Spring of 2017. The VSH queen was sourced from the USDA-ARS Honey Bee Breeding Laboratory in Baton Rouge, where queens are selected based on suppression of mite reproduction. The HYG queen was sourced from the well-established breeder Jeff Hull (Minnesota) and was selected based on removal of>95% freeze-killed brood. The UNS queen was sourced from Triad Bee Supply which obtains their queens from Gardner Apiaries in Baxley, Georgia. For each experimental hydrocarbon, a control hydrocarbon of similar size and structure, but not known to be a component of the honey bee’s cuticular hydrocarbons, was also tested. Hydrocarbons (Z10-C_33_, Z16-C_32_, Z6-C_15,_ and Z7-C_15_) were synthesized by Z-selective Wittig reactions between the appropriate aldehydes and phosphonium salts, or by Z-selective olefin metathesis reactions. Crude products were purified in two steps, by flash vacuum chromatography on silica gel, eluting with hexanes, followed by recrystallization from acetone at ~4 °C for longer chain compounds, or ~−20 °C for shorter chain compounds. Synthesis is described in further detail, below. Dilutions in hexane of 0.1%, 0.3%, and 1.0% (wt/vol) were prepared for each hydrocarbon. The lowest dilution is equivalent to that previously reported^49^, and higher dilutions were chosen to approximate dose effects on a logarithmic scale. All sample collections and analyses were conducted at the University of North Carolina at Greensboro.

### Synthesis of alkenes tested in bioassays

#### Synthesis of Z6-C_15_

A solution of 1-decyne (5.52 g, 40 mmol) and ~50 mg triphenylmethane indicator in dry THF under Ar was cooled to ~−15 °C in an ice/acetone bath, and butyllithium (2.6 M in hexanes) was added dropwise until the solution turned pink. An additional 15.4 ml of butyllithium solution (40 mmol) was then added over 30 min, and the resulting solution was warmed to room temperature and stirred 1 h. Powdered NaI (0.6 g, 4 mmol) was then added, followed by dropwise addition of bromopentane (3.93 g, 26 mmol). The mixture was heated to reflux and stirred 22 h, then cooled and quenched with 1 M aqueous NH_4_Cl solution, and extracted with hexane. The hexane layer was washed with saturated NaHCO_3_ and brine, then dried and concentrated. The residue was purified by Kugelrohr distillation, taking a forerun of the excess 1-decyne (oven temp <40 °C, 0.05 mm Hg), then changing the collection bulb and collecting the desired product (2.8 g, bp~60 °C, 0.05 mm Hg).

The distilled product was flushed through a plug of silica gel with hexane and into a 200 ml round-bottomed flask with a magnetic stir bar. Lindlar catalyst (150 mg) and quinolone (1.5 ml) were added, and the flask was sealed and flushed with nitrogen, then hydrogen. With the sealed flask attached to a gas burette filled with hydrogen, stirring was then started, resulting in uptake of ~310 ml of hydrogen, at which point uptake virtually ceased. The flask was flushed with nitrogen, and the mixture was filtered through a plug of celite, rinsing well with hexane. The resulting solution was washed twice with 1 M HCl, then dried and concentrated. The residue was flushed through a pad of silica gel with hexane, then Kugelrohr distilled (2.82 g, bp~60 °C, 0.05 mm Hg). Because the resulting product was contaminated with about 4% of the alkyne starting material, a portion (1.2 g) was repurified by vacuum flash chromatography on silica gel in a 60 ml Buchner funnel. The silica was prewetted with hexane, then the impure alkene was loaded as a hexane solution, and the column was eluted with 5 ×30 ml hexane. Z6-C_15_ eluted cleanly in fractions 1 and 2, with the alkyne eluting in fractions 4 and 5. EI Mass spectrum (*m/z*, abundance): 210 (53, M^+^), 182 (3), 168 (1), 154 (2), 140 (4), 125 (15), 111 (41), 97 (79), 84 (42), 83 (90), 70 (69), 69 (100), 57 (59), 56 (59), 55 (91), 43 (42), 41 (56).

#### Synthesis of Z7-C_15_

Z7-C_15_ was made in analogous fashion from 1-octyne and 1-iodoheptane. EI Mass spectrum (*m/z*, abundance): 210 (41), 182 (3), 168 (1), 154 (2), 140 (4), 125 (13), 111 (38), 97 (76), 84 (43), 83 (89), 70 (69), 69 (100), 57 (59), 56 (63), 55 (94), 43 (63), 41 (64).

#### Synthesis of Z16-C_32_

1-Heptadecene (2.38 g, 10 mmol; GFS Chemicals, Powell OH) was flushed through a plug of silica gel with hexane, then concentrated and transferred to a dry 3-neck flask. The flask was flushed thoroughly with Ar while stirring and warming to 45 °C. Grubbs catalyst C675 (190 mg, 0.25 mmol; gift from Materia Inc., Pasadena CA) was added in one portion, and the mixture was stirred 3 h at 45 °C under a slow flush of nitrogen to remove the ethylene formed. The mixture was then cooled, diluted with hexane, and flushed through a plug of silica gel with hexane. The resulting semicrystalline residue was Kugelrohr distilled to 105 °C (0.05 mm Hg) to remove unreacted 1-heptadecene, and the residue was recrystallized from hexane at −20 °C. The resulting white crystals (1.04 g) were 99.6% chemically pure by GC. The *Z/E* ratio (>99% *Z*) was checked by epoxidation of a sample with *meta*-chloroperbenzoic acid in methylene chloride, followed by GC-MS; the *trans*-epoxide eluted before and was completed separated from the *cis*-epoxide. EI Mass spectrum (*m/z*, abundance): 448 (20), 420 (2), 376 (1), 362 (1), 334 (1), 320 (1), 306 (2), 292 (2), 278 (2), 264 (2), 250 (2), 236 (3), 222 (3), 210 (3), 196 (4), 181 (5), 167 (7), 153 (10), 139 (16), 125 (34), 111 (61), 97 (100), 85 (37), 83 (82), 71 (50), 69 (56), 57 (74), 55 (52), 43 (54), 41 (25).

#### Synthesis of Z10-C_33_

Triflic anhydride (2.2. ml, 12 mmol) was added dropwise to a slurry of docosanol (3.26 g, 10 mmol), pyridine (0.8 ml, 10 mmol), and ~50 mg dimethylaminopyridine catalyst in 50 ml methylene chloride, cooling as necessary to keep the reaction temperature <25 °C. When the addition was complete the mixture was stirred 1 h at room temperature, producing a pale brown, slightly cloudy solution. The solution was diluted with 100 ml hexane, and filtered through a pad of silica gel, rinsing the filter pad with 2:1 hexane in methylene chloride. The resulting clear solution was concentrated to a white solid which was taken up in ether and used immediately.

Butyllithium (2.24 M in hexanes) was added to an ice-bath cooled solution of 1-undecyne (2.28 g, 15 mmol) and ~50 mg triphenylmethane indicator in 50 ml dry THF under Ar until a pink color persisted (~7 ml, 15.7 mmol). The solution was stirred for 1 h, then the ether solution of the triflate was added dropwise at 0 °C, and the mixture was warmed to room temperature and stirred overnight. The reaction was then quenched with saturated aqueous NH_4_Cl, and extracted with hexane. The hexane layer was washed with brine, dried, and concentrated. The residue was flushed through a pad of silica gel with hexane, then Kugelrohr distilled (oven temp ~50 °C, 0.2 mm Hg) to remove excess 1-undecyne. The solid residue was then recrystallized from 50 ml acetone, warming to solubilize the product, then cooling to room temperature, yielding the alkyne product as a single peak (1.76 g), with additional impure alkyne in the filtrate.

The alkyne (1.7 g) was taken up in 30 ml hexane, and quinoline (0.75 ml) and Lindlar catalyst (75 mg) were added. The reaction flask was sealed and flushed sequentially with nitrogen, then hydrogen, then connected to a gas burette filled with hydrogen. The mixture was stirred until hydrogen uptake ceased. After flushing with nitrogen, the mixture was filtered through celite, most of the quinoline was removed under high vacuum, and the residue was recrystallized from 30 ml of hot acetone, after cooling to 4 °C. The resulting white solid was still contaminated with quinoline, and so a hexane solution was flushed through a plug of silica gel with hexane. After concentration, the residue was recrystallized again from acetone, yielding the alkene as a white solid (1.64 g, 98.7% pure by GC). EI Mass spectrum (*m/z*, abundance): 462 (12), 434 (1), 390 (1), 376 (1), 362 (1), 348 (1), 334 (1), 320 (1), 306 (1), 292 (1), 278 (1), 264 (2), 250 (2), 236 (1), 222 (2), 208 (3), 195 (3), 181 (4), 167 (6), 153 (9), 139 (14), 125 (30), 111 (54), 97 (97), 85 (37), 83 (84), 71 (54), 69 (69), 57 (100), 55 (74), 43 (90), 41 (43).

#### Hygienic *response to treatment of pupae with* Z10-C_33_

This experiment was conducted using one VSH, one HYG, and one UNS colony. To obtain a same-age cohort of honey bee brood, the locations of uncapped brood cells containing 5^th^ instar larvae were marked using a permanent marker on transparent plastic sheets secured above experimental cells with thumbtacks. Combs containing experimental cells were placed back into the colony and recollected within 8 h. Cells capped within that time were marked for experimental use, and frames were returned to their respective colonies. On day 6 post-capping, experimental cells were opened by cutting and lifting one side of the cell cap with a razor blade. The pupa underneath received either no treatment, or treatment with either 1 μL of hexane or with 1 μL of 1.0% Z10-C_33_ in hexane. Cells were then resealed by gently pressing the cap against the cell wall with the side of a razor blade, and frames were returned to their respective colonies. Uncapping and removal of brood in experimental cells were recorded at 4 and 24 h after the frame was reintroduced. Sample sizes were 40 cells per treatment for UNS and HYG colonies, and 50 cells per treatment for the VSH colony.

#### *Effects of treatment of pupae with* Z10-C_33_*on development*

This experiment was conducted using the same three VSH, HYG, and UNS colonies. As described above, capped cells containing brood 6-d post-capping were carefully opened, and the brood inside received either no treatment, or treatment with either 1 μL of hexane, or 1 μL of 1.0% Z10-C_33_ in hexane. Pupae were then removed from the brood comb using flexible-tipped forceps, and gently placed onto fan-folded filter paper in Petri dishes. Petri dishes were placed in an incubator maintained at 34 °C and 50% RH. Brood was examined for injury (dark pigmentation) after 48 h in the incubator, and any injured brood were discarded. On the day after expected emergence, brood were examined for normal development, defined by typical adult pigmentation and shape with proper wing development. Any deviation from this was considered “deformed” and used to calculate developmental success. Brood sample sizes were 35, 29, and 27 individuals per treatment for VSH, HYG, and UNS colonies, respectively.

### Hygienic response to wax cap treatment

We tested effects on hygienic behavior of application of test compounds to wax caps of brood cells. Hygienic assays were conducted by applying hexane, 0.1%, 0.3%, or 1.0% dilutions of Z10-C_33_, Z6-C_15_, or appropriate controls (Z16-C_32_ and Z7-C_15_, respectively) in hexane to capped brood cells in a VSH colony. For each assay, 2 mL of solution were applied to a circular area of capped honey bee brood. Similar to the established freeze-killed brood assay^68^, the treated area was isolated using a piece of PVC pipe (7.5 cm inner diameter, approximately 8 cm long). Chemicals were applied using an H-100D Single Action airbrush and compressor (Paasche, Kenosha, WI), modified with glass bottles fitted with glass tubing for this application. For each assay, 2 mL of the solution were added to the bottle immediately before application to wax caps. Thus, 0.1%, 0.3%, and 1.0% solutions deposited approximately 45, 136, and 453 µg hydrocarbon/cm^2^ of capped honey bee brood, respectively. Capped cells were counted directly after treatment, and frames were returned to the colony. After 24 h, frames were recollected, and capped cells in the treated region were recounted. For comparisons of Z10-C_33_, Z16-C_32_, and hexane, sample sizes were 8, 6, and 3 replicates, respectively. For comparisons of Z6-C_15_, Z7-C_15_, and hexane, sample sizes were 12, 6, and 5 replicates, respectively. Because brood availability was limited, priority was given to replication of Z10-C_33_ and Z6-C_15_ assays, followed by assays of the structural controls Z16-C_32_ and Z7-C_15_. Assay scores were determined by dividing the total number of uncapped and removed cells after 24 h by the total number of capped cells in the circular assay area at the beginning of the assay.

### Effects of wax cap treatment on development

Capped VSH brood cells in a 7.5 cm diameter circular area were left untreated (control) or treated with 2 mL of hexane, 0.1%, 0.3%, or 1.0% Z10-C_33_ or Z6-C_15_ solutions in hexane with the airbrush, as described above. After 30 min, white-eye pupae (aged 5-6 days post-capping) were removed from the brood comb using flexible-tipped forceps, and gently placed onto filter paper in Petri dishes held in an incubator (34 °C, 50% RH). Brood was examined for injury (dark pigmentation) after 48 h in the incubator, and any injured brood were discarded. On the day after expected emergence, brood were examined for normal development, as described above. Any deviation from this was considered “deformed” and the proportion of successful development calculated. Brood sample size was 40 pupae per treatment.

### Comparison of freeze-killed brood (FKB) and Z10-C_33_-treatment assays

Hygienic responses to Z10-C_33_ wax cap treatment and FKB assays were compared in ten colonies, including 2 HYG, 2 VSH, and 6 UNS. For the chemical assay, 1.0% Z10-C_33_ was applied to wax caps as described above, and any uncapping or removal of a cell after 24 h was counted as a cell targeted by hygienic behavior. The percentage of such cells among all initially capped cells was calculated for the assay score, as above. For FKB assays, a 7.5 cm diameter PVC tube was placed on a section of capped pupae aged 3-10 d post-capping, and brood in the assay area were frozen using liquid nitrogen. Frames were returned to their colony of origin, and after 24 h, all cells within the test area that still contained any pupae were counted and recorded, according to standard practice^68^. Assay scores were determined by dividing the total number of cells containing any pupae after 24 h by the total number of capped cells in the circular assay area at the beginning of the assay and subtracting this number from 1.

### Statistical analyses

Pearson’s Chi-square analysis with Bonferroni correction was used to test effects of pupal treatment on hygienic responses in VSH, HYG, and UNS colonies. All analyses were comparisons of manipulated (uncapped or removed) versus non-manipulated (capped) cells. Pearson’s Chi-square analysis with Bonferroni correction was also used to test effects of pupal treatment on the numbers of successfully and unsuccessfully developing VSH, HYG, and UNS brood. Two-way ANOVAs with Bonferroni-corrected post-hoc comparisons were used to test effects of chemical type and chemical concentration on hygienic response to wax cap treatments. Pearson’s Chi-square analysis with Bonferroni correction was used to test effects of wax cap treatment on the development of VSH, HYG, and UNS brood. A Pearson’s correlation coefficient was calculated to test for a positive correlation between hygienic responses to Z10-C_33_ and FKB assays across colonies, and a one-tailed p-value is reported. Chi-square analyses were calculated based on raw data, while assay scores and related analyses were based on percentages. All statistics were performed using IBM SPSS Statistics, Version 25.

## Data Availability

The datasets generated during and/or analyzed during the current study are available from the corresponding author upon request.
